# Digital pathology all stars

**DOI:** 10.1016/j.jpi.2022.100125

**Published:** 2022-07-16

**Authors:** Albino Eccher, Ilaria Girolami, Aldo Scarpa

**Affiliations:** a Department of Pathology and Diagnostics, University and Hospital Trust of Verona, Verona, Italy; b Department of Pathology, Provincial Hospital of Bolzano (SABES-ASDAA), Bolzano-Bozen, Italy; Lehrkrankenhaus der Paracelsus Medizinischen Privatuniversität; c Department of Pathology and Public Health, University and Hospital Trust of Verona, Verona, Italy

**Keywords:** Digital pathology, Artificial intelligence, Telemedicine, Telepathology

## Abstract

Digital pathology plays an important role in accelerating the progression of healthcare and the potential benefits of adopting digital technologies have been solidly established**.** Despite this, real-world data suggest that a fully digital approach to the histological workflow has been implemented in a minority only of pathology laboratories. The e-learning event “Digital Pathology All Stars” was conceived by the University and Hospital Trust of Verona and comprised traditional lectures made by well-recognized experts in Digital Pathology from all over the world. The meeting aimed to promote the exchange of knowledge to support and strengthen digital pathology adoption and implementation.

## Introduction

The community of pathologists is making a great effort for the implementation of digital pathology (DP) into clinical practice. Digital pathology plays an important role in accelerating the progression of healthcare and the potential benefits of adopting digital technologies have been solidly established.^1^, ^2^, ^3^, ^4^ In addition, useful guidelines for implementation and validation have been developed and improved.^5^^,^^6^ The e-learning event “Digital Pathology All Stars” was conceived by the University and Hospital Trust of Verona where traditionally the room for improvement of digital pathology has always been a primary goal. The meeting was organized employing Zoom videoconferencing software (Zoom Video Communications, Inc., San Jose, CA, USA) on May 6, 2022, and comprised of traditional lectures and interactive sessions, with the involvement of 9 lecturers and 6 moderators. The title of the meeting was borrowed from NBA basketball in which the annual All-Star brings together the best profiles of the championship. The meeting aimed to promote the exchange of knowledge with internationally recognized opinion leaders on DP.

## Meeting overview

**Fig. 1:**
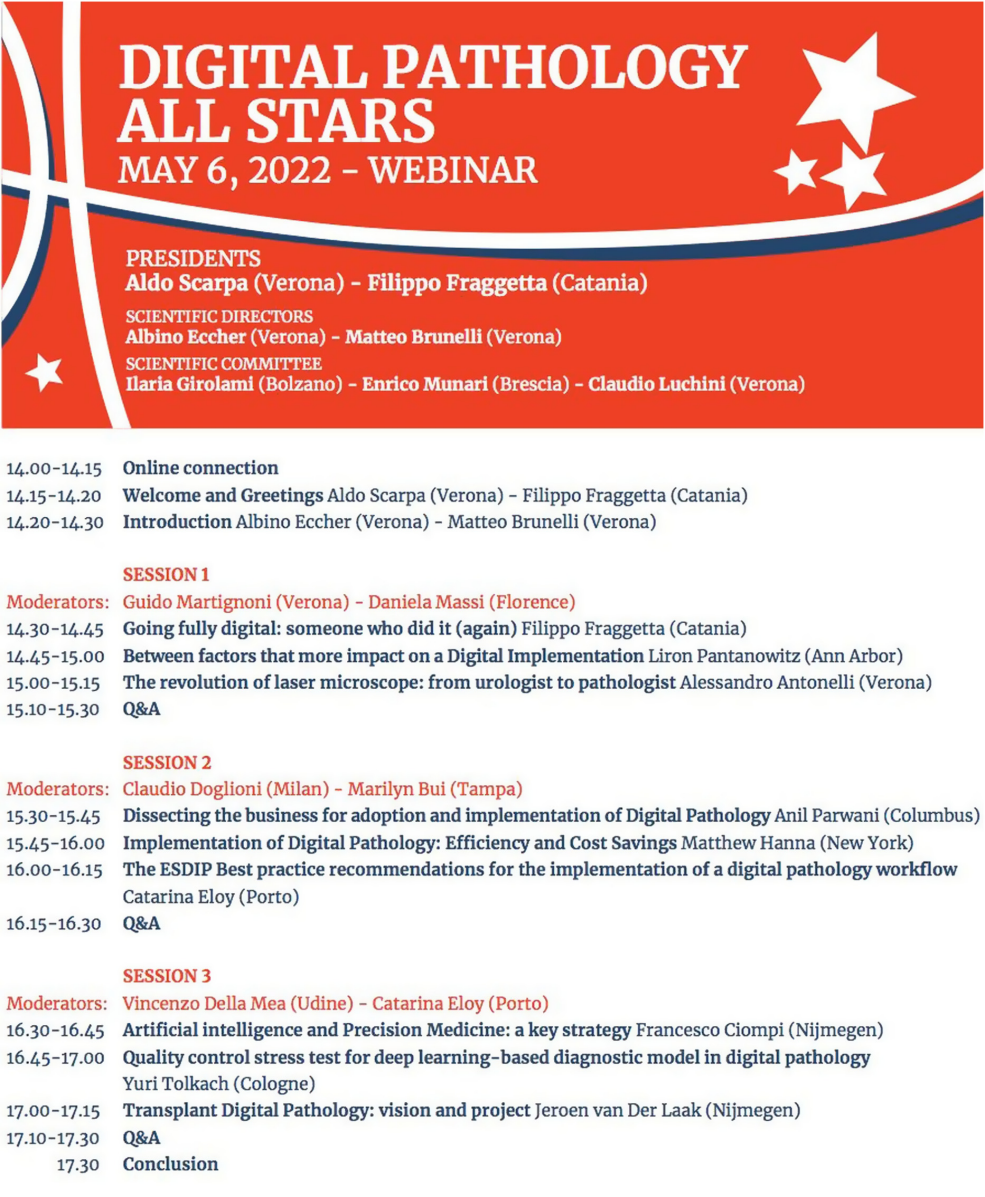
Meeting overview with scientific program.

The symposium was divided into 3 scientific sessions with invited talks forming the backbone of the program (Fig. 1). Moderators of the Session 1 were Guido Martignoni (Verona, Italy) and Daniela Massi (Florence, Italy), Claudio Doglioni (Milan, Italy) and Marilin Buy (Tampa, USA) for Session 2, and Catarina Eloy (Porto, Portugal) and Vincenzo Della Mea (Udine, Italy) for Session 3. The titles of the talks were as follows:

Session 1: Going fully digital: someone who did it (again) - Filippo Fraggetta (Caltagirone, Italy), Between factors that more impact on a Digital Implementation - Liron Pantanowitz (Ann Arbor, USA), The revolution of laser microscope: from urologist to pathologist - Alessandro Antonelli (Verona, Italy).

Session 2: Dissecting the business for adoption and implementation of Digital Pathology - Anil Parwani (Columbus, USA), Implementation of Digital Pathology: Efficiency and Cost Savings - Matthew Hanna (New York, USA), The ESDIP Best practice recommendations for the implementation of a digital pathology workflow Catarina Eloy (Porto, Portugal).

Session 3: Artificial intelligence and Precision Medicine: a key strategy Francesco Ciompi (Nijmegen, Holland), Quality control stress test for a deep learning-based diagnostic model in digital pathology - Yuri Tolkach (Cologne, Germany), Transplant Digital Pathology: vision and project - Jeroen van Der Laak (Nijmegen, Holland).

## Sessions highlights

Filippo Fraggetta shows its commitment to digital implementation, in compliance with the ESDIP (European Society of Digital and Integrative Pathology) guidelines. Indeed, despite the potential benefits of adopting digital technologies have been solidly established, real-world data suggest that a fully digital approach to the histological workflow has been implemented in a minority only of pathology laboratories. At the same time, however, some of the laboratories are trying to switch to a digital workflow. Some of the barriers we have faced have been: validation of the systems including regulatory approval, interfaces between whole slide images (WSI) systems and the electronic medical record, current laboratory workflow, cost, and pathology willingness to go digital.^7^ The talk offers a detailed narrative on how a well-thought-out approach to digital is the key to reliable and successful project planning.

**Fig. 2:**
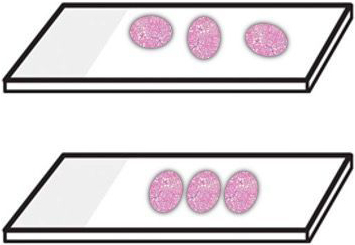
Optimal tissue section arrangement. The top slide shows pieces of tissue spread widely across the slide and in different orientations, which is to be avoided. The bottom slide shows ideal placement with minimal space between tissue pieces that are all oriented the same way.

Liron Pantanowitz provides a comprehensive overview of the current impact of Pre-Imaging Factors on DP workflow which encompasses an imaging phase (e.g. scanning), and subsequent utilization of acquired images for various applications (e.g. primary diagnosis, image analysis, etc). To date, limited effort has been devoted to the pre-imaging phase compared to scanning and imaging applications. Tweaking pre-imaging steps are necessary for the successful deployment of DP because poorly produced pathology glass slides are likely to lead to low-quality digital slides. Before scanning, the key factors that will need to be optimized are specimen fixation, grossing, tissue processing, embedding, microtomy, staining, coverslipping, and barcoding of slides. For example, it is important to ensure there is constant section thickness as thick sections can affect image resolution quality.^8^ Moreover, thin tissue sections cause greater stain variability. In addition, the arrangement of tissue pieces on the slide is equally important. Embedding samples all in a row instead of scattered all over the slide results in less empty space between tissue fragments, which in turn decreases scan time, reduces the image file size, and the time taken by the pathologist for viewing (Fig. 2). According to guidelines for whole slide imaging (WSI) validation for diagnostic purposes published by the College of American Pathologists (CAP), it is imperative that the validation process confirm that all the material on a glass slide to be scanned is included in the digital image. One mechanism to satisfy this requirement is to use a device (e.g. BlocDoc) to rapidly capture digital images of all paraffin tissue blocks. This allows all digitized slides to be matched and tracked with their corresponding blocks.^9^ The barcode on the block is used to upload these images directly into the case within the laboratory image system (LIS). For high-volume scanning to succeed and have all digital slides linked to the correct cases in the LIS, all glass slides must ideally have a barcode. However, inferior or even slightly imperfect barcodes are the Achilles’ heel of digital pathology because this is a frequent cause of scan failures, where unmatched slides linger in cyberspace instead of being properly linked to the LIS. This accordingly requires clear legible barcodes, as well as regular maintenance of label printers. Since etched slides may cause fewer barcode errors these slides may yield more successful scanning. Currently, it is unclear if quality control (QC) measures should be performed before or after scanning to ensure flawless digitized slides. Some laboratories clean all their slides before scanning to make sure they are free of debris or pen markings and check that coverslips are intact and do not overhang the slide. However, such manual QC steps are laborious and not feasible for scaling slide digitization. Instead, QC steps could be performed post-scanning by having technologists pan all or only a small subset of digital slides at low magnification for imaging artifacts (e.g. missing tissue, out-of-focus areas). More promising, there have been several machine learning tools developed (e.g. blur detection, focus heatmap, tissue fold detection, stain correction), that could be leveraged for automating QC of scanned slides.

In his talk, the urologist Alessandro Antonelli tracks and analyzes the results of the application of confocal laser microscope in uropathology, in contrast to standard pathologic analysis of fixed tissue with hematoxylin and eosin. To decrease the learning curve and facilitate clinical studies, the development of a computer-based smart atlas may be beneficial through collaborative efforts among urology, pathology, and imaging scientists. With efforts underway to increase the usability of this tool, the laser microscope promises to achieve better surgical outcomes and more effective management for patients.

The objectives of the talk of Anil Parwani are to give critical insights on how DP can increase productivity and revenues, improve quality in medicine, and reduce costs. From a healthcare quality standpoint, DP can improve analysis, reduces errors, and offers better views as opposed to traditional pathology. From a productivity standpoint, DP can improve workflow, reduces turnaround time, and allow for more innovation. Hospital and diagnostic labs are becoming the biggest adopters of DP tools and vendors can boost their chances of approval and commercialization by staying focused on cost optimization for clinicians. Furthermore, global pathology demands show a worldwide shortage of pathologists. The global market for DP is expected to grow by over 12% per year. While North America will continue to dominate the DP market over the next decade, the Asia-Pacific market will have the highest growth.^10^ In the USA, there is currently no billable code for telepathology; codes that can be used include consultation and report on referred slides prepared elsewhere ($ 104 80), consultation and report on referred material requiring preparation of slides ($ 131 71), consultation, comprehensive, with the review of records and specimens, with report on referred material ($ 191 29). The key value driver is to focus on data generated by DP with reference to cost optimization, efficiency gains, and clinical accuracy.^11^

Matthew Hanna emphasizes how Pathology is undergoing a digital transformation where early adopters have shown increases in efficiency and cost savings in clinical operations. By having leadership support to initiate the journey of digital transformation, the process can begin. The laboratory will require pre-analytic components and integrations to be implemented before DP, however, this will also help ensure a lean deployment is had with additional efficiencies achieved. Then the scanning hardware and software allow laboratories to save time, money, and personnel resources by capturing the physical glass media into a digital whole slide image. While there is an upfront capital cost, laboratories show decreased time for courier services, transportation/shipping costs, consultation case review, and overall better experience for those laboratories who have embraced the technologies.^12^ Furthermore, machine learning systems will further enhance pathologists’ productivity through clinical decision support and assertive workflows.

Catarina Eloy focuses on the ESDIP best practice recommendations for the implementation of a DP workflow which is a complementary document of other well-known guidelines and recommendations on the topic, that is specifically devoted to the pre-scanner processes as well as their quality control. To have a team-based and linear approach is essential to revisit the basic operations at the pathology laboratory, adjusting each workstation to the new methodology, so-called digital pathology. Pathology laboratories that spend their efforts optimizing the pre-scanner processes, adapting them to the final goal of producing a high-quality whole slide image, guarantee the sustainability of the DP workflow, and establish a solid basis for the implementation of artificial intelligence tools. In this talk, a democratization of the digital transformation is defended as well as the establishment of specific educational programs in each laboratory to cope with the patients' demands and current world challenges.

In his talk, Francesco Ciompi provides examples of how computational pathology powered by artificial intelligence can effectively improve diagnostic workflows in DP and support cancer treatment planning. Existing Artificial Intelligence (AI) technology can automate mitotic count in breast cancer, providing visual feedback to pathologists and reducing interobserver variability at the mitotic count. The talk focus then on the use of AI immuno-oncology applications where AI can support biomarker quantification such as the tumor proportion scoring based on PD-L1 staining, the detection of tertiary lymphoid structures, and their germinal centers, and the quantification of tumor-infiltrating lymphocytes, towards fully-automated quantification of composite biomarkers.^13^^,^^14^ Finally, the idea of using AI to learn to predict patient-level end-points is discussed, such as survival or treatment response, directly from the raw data. When equipped with tools to visualize the salient regions attended by the model, AI-based models might serve as a potential tool for pattern discovery.

Yuri Tolkach faces heterogeneity of histological slides concerning staining, sections' thickness, and artifacts arising during tissue processing, cutting, staining, and digitization. In a recent paper, using 6 datasets from 4 different institutions digitized by different scanner systems, they systematically explore artifacts' influence on the accuracy of the pre-trained, validated, deep learning-based model for prostate cancer detection in histological slides. Results provide evidence that any histological artifact dependent on severity can lead to a substantial loss in model performance. Stress-testing of diagnostic models using synthetically generated artifacts might be an essential step during clinical validation of deep learning-based algorithms.^15^

Lastly, the talk of Jeroen Van Der Laak turns on transplants. Indeed, while most studies focus on oncology, preliminary data also suggest an important future role for AI in evaluating renal transplant biopsies. AI has the potential to yield objective, reproducible, and quantitative data, supporting the early detection of pathological processes that may ultimately lead to graft loss. Our previous research has shown how AI can successfully delineate (‘image segmentation’) anatomical structures (possibly displaying pathological changes) in renal biopsies.^16^ Also, we developed techniques to automatically detect immunohistochemically stained lymphocytes. In a recently started project, we will bring together existing technologies, to arrive at fully automated scoring of many Banff features. Developed AI will be validated in a multicenter setting against graft survival and will subsequently be available through web technology for use in subsequent validation studies and research use.

## Conclusion

Despite the unavoidable benefits of adopting digital technologies, real-world data suggest that a fully digital approach to the histological workflow has been implemented in a minority only of pathology laboratories. The “Digital Pathology All Stars” successfully brought together recognized experts which reached significant results thanks to their effort and proven ability to innovate and accomplish advancement in digital pathology.

## Declaration of Competing Interest

The authors declare that they have no known competing financial interests or personal relationships that could have appeared to influence the work reported in this paper.
